# Full-length carbon fiber insole alters lower leg muscle activity in patients with midfoot arthritis

**DOI:** 10.1186/1757-1146-7-S1-A117

**Published:** 2014-04-08

**Authors:** Tae Im Yi, Ji Hye Hwang, Tae Hee Yoon, Ji Yang, Jung Hyun Kim

**Affiliations:** 1 Department of Physical and Rehabilitation Medicine, Bundang Jesaeng General Hospital, Seohyeon-dong, Bundang-gu, Seongnam 463 - 774, Korea; 2 Department of Physical and Rehabilitation Medicine, Sungkyunkwan University School of Medicine, Samsung Medical Center, 50 Irwon-dong, Gangnam-gu, Seoul 135-710, Korea; 3 Center for Clinical Medicine, Samsung Medical Center, 50 Irwon-dong, Gangnam-gu, Seoul 135-710, Korea

## Objective

This study aimed to evaluate the effect of the full-length carbon fiber (FCF) insole on the lower leg muscle activity and the GRF during walking in patients with unilateral midfoot arthritis.

## Methods

Fifteen patients with midfoot arthritis aged between 20 and 60 years participated in the study. Foot function index (FFI) was assessed to evaluate pain before and after the walking activity. Lower leg muscle activity, GRF and gait cycle time during walking were measured under two conditions: “shoe only”, and “shoe with FCF insole”. Electromyography (EMG) activities of the tibialis anterior (TA), gastrocnemius medialis (GCM) and soleus muscles in the involved leg were assessed by multi-channel telemetry EMG. Simultaneously, the GRF was measured under both conditions. The gait cycle was divided into four different phases including loading response (LR), mid-stance (MS), terminal stance (TS), and pre-swing (PS).

## Results

With reference to the pain subscale of the FFI, we divided the patients into “reported pain reduction” and “reported no pain reduction” groups. While walking under the shoe with FCF insole condition, significant reductions of the TA muscle activity were observed in the LR phase only in the group that reported pain reduction (p = 0.028). Contrastingly, in the group that reported no pain reduction, the TA muscle activity showed no difference between both conditions (p = 0.237). As for the measurement of the GCM muscle activity, under the shoe with FCF insole condition, a significant reduction was observed in the TS and PS phases only in the group that reported pain reduction (p = 0.046 and p = 0.046). However, in the group that reported no pain reduction, there was no significant reduction of GCM muscle activity in any of the gait phases under either the shoe with FCF insole condition or the shoe only condition (p > 0.05). Neither group reported any statistically significant reduction in the GRF or gait cycle time (p > 0.05).

## Conclusion

Symptomatic improvement in patients with midfoot arthritis during walking with the FCF insole was accompanied by reduced TA muscle activity in the LR phase along with reduced GCM muscle activity in the TS and PS phases. However, walking with the FCF insole was not effective in reducing the GRF or gait cycle time. From a clinical perspective, these findings suggest that prescription of the FCF insole can be a viable alternative to other non-operative treatments in patients with midfoot arthritis.

